# ﻿*Orobancheandryalae* (Orobanchaceae): a new species from the Canary Islands

**DOI:** 10.3897/phytokeys.252.141300

**Published:** 2025-02-20

**Authors:** Chris J. Thorogood, Matías Hernández González, Fred J. Rumsey, Jorge Alfredo Reyes-Betancort

**Affiliations:** 1 University of Oxford Botanic Garden, Rose Ln, Oxford OX1 4AZ, UK University of Oxford Botanic Garden Oxford United Kingdom; 2 Department of Biology, University of Oxford, South Parks Road, Oxford OX1 3RB, UK University of Oxford Oxford United Kingdom; 3 Asociación Ambiental Arrecife Natura, C/ Guillermo Toledo Duchemín, 13. 35500, Arrecife, Las Palmas, Spain Asociación Ambiental Arrecife Natura Las Palmas Spain; 4 Natural History Museum, Cromwell Rd, South Kensington, London SW7 5BD, UK Natural History Museum London United Kingdom; 5 Jardín de Aclimatación de La Orotava, Instituto Canario de Investigaciones Agrarias (ICIA), C/ Retama 2, 38400 Puerto de la Cruz, S/C de Tenerife, Spain Instituto Canario de Investigaciones Agrarias (ICIA) Tenerife Spain

**Keywords:** *Andryala*, broomrape, endemism, Macaronesia, parasitic plant

## Abstract

A new species of *Orobanche* is described from the Canary Islands. The plant belongs to the complicated Subsection Minores and has a distinct combination of morphological features, ecology, and host specificity. The markedly cernuous corolla, high filament insertion and colouration distinguishes *Orobancheandryalae* from closely related species with which it has been previously confused, and which do not occur in the Canary Islands, including O.amethysteasubsp.castellana and *O.calendulae*; these features remain stable in cultivation. *Orobancheandryalae* appears to be parasitic almost exclusively on *Andryala* spp., and occurs on thermophilus volcanic substrates in northern Lanzarote, northwest Tenerife and Jandía in Fuerteventura. An updated key to the *Orobanche* of the Canary Islands is presented, including the new taxon we describe here.

## ﻿Introduction

Broomrapes (genus *Orobanche* L., family Orobanchaceae Vent.) are taxonomically challenging. They are holoparasitic so devoid of functional leaves and other diagnostic characteristics, and many of the characters useful for identification such as stigma and corolla colour, are lost when dry. Herbarium specimens are often determined incorrectly and have inadequate field notes. Particularly challenging are closely related species in the Subsection Minores Teryokhin: a group of similar, small-flowered species (corollas typically < 20 mm) (see e.g. El Mokni et al. 2015). Beck-Mannagetta (1930) in his comprehensive monograph of the genus, listed over 345 binomials belonging to this group (to which he referred as the Grex *Minores*) and recognised 20 species, 20 varieties, and a total of 69 forms. Existing phylogenies have not successfully resolved taxa in the Subsection Minores. Moreover, genetically distinct host races can be obscured by cryptic morphology (Thorogood et al. 2009). In the absence of a comprehensively sampled and well-resolved phylogeny, a combined approach that considers ecology, host range, and stable morphological features (such as filament insertion and corolla dorsal line) is needed to tease apart taxa in this complicated group.

There are at least seven species of *Orobanche*, and a further seven species of the related genus *Phelipanche* Pomel, recorded to occur in the Canary Islands (BIOTA 2024), but a detailed taxonomic assessment for the area is absent and necessary. Within the Subsection Minores, five species have been recorded to occur in the region: *O.minor* Sm., *O.calendulae* Pomel, *O.artemisiae-campestris* Vaucher ex Gaudin (as cf. *O.loricata* – doubtfully, probably in error for the species under consideration) and *O.amethystea* Thuill., together with *O hederae* Duby (also considered, based on molecular evidence, to belong to the similar Subsection Hederae Teryokhin). The most widespread of these is *O.minor* which occurs across Europe and the Mediterranean Basin, and is widely naturalised in temperate regions across the globe. This species tends to occur in urban and ruderal sites on all the Canary Islands and may be introduced to the region. *Orobancheminor* is lax, small-flowered (corolla 10–18 mm), and purplish in colour; it grows on a wide variety of hosts from 16 orders (Thorogood et al. 2009). *Orobanchehederae* occurs in damp woods on *Hedera* – but once also reported to grow on *Geraniumreuteri* Aedo & Muñoz Garm. (Gilli 1980) - on the western islands (Tenerife, La Gomera, La Palma) and is easily distinguished by its yellow stigma (in other species the stigma is variable but typically pinkish, reddish, purplish), corollas with a distinct constriction behind the corolla mouth, and acute and lacerate corolla lower lip lobes. *Orobancheamethystea* is widespread across Europe and is usually parasitic on *Eryngiumcampestre* L., but has been recorded on other hosts including various other Apiaceae Lindl., and even *Digitalis* Tourn. ex L. (Plantaginaceae) (Chater and Webb 1972). It is superficially similar to *O.minor* but more robust, with a larger corolla (15–25 mm) that is rather sharply inflected near the base, rather flat along the dorsal line, and geniculate near the tip (rather than with a smoothly-curved dorsal line), with a somewhat 2-lobed upper lip, and high filament insertion. *Orobancheamethystea* has long been recorded to occur on Lanzarote and Tenerife (Reyes-Betancort et al. 2000; Padrón-Mederos et al. 2009); the subspecies reported there is subsp. castellana (Reut.) Rouy which differs from the type subspecies in lacking filiform calyx teeth, pinkish-brown (rather than violet) flowers, and the upper corolla lip being less conspicuously divided. However, these plants identified as *O.amethystea* in the Canary Islands lack nearly all the features associated with that species. They also differ markedly from *O.minor* with which it co-occurs, but in a different niche (ruderal sites). The plant in question has only been recorded to parasitize *Andryalaperezii* M.Z.Ferreira, R.Jardim, Alv.Fern. & M.Seq. (Ferreira et al. 2014), endemic to Lanzarote and Fuerteventura, and to a lesser extent, on *Andryalapinnatifida* Aiton on Tenerife (a species that occurs on all the Canary Islands) - an unusual but not unquestionable host range for *O.amethystea*; the genus *Andryala* is also the host range of *O.almeriensis* A.Pujadas (González and Pujadas Salvà 1995), which also belongs to the Subsection Minores; however this too, is morphologically dissimilar to the material in question (Table 1). The latter author (JARB) has also observed the taxonomic entity in question apparently growing on *Asteriscussericeus* (L.f.) DC. once, on Pico de la Zarza, the highest peak of Fuerteventura. This population needs revisiting.

**Table 1.** *Orobancheandryalae* and closely related taxa.

	*O.andryalae*	*O.calendulae*	*O.almeriensis*	*O.amethystea*	*O.minor*
Host	*Andryalaperezii*	*Calendulasuffruticosa*	*Andryalaragusina*	*Eryngiumcampestre*; various Apiaceae; (rarely other hosts)	Numerous hosts, especially Fabaceae, Asteraceae, Apiaceae
Stem	Slender, pale reddish-orange; flowers ± lax, over the upper part of the stem	Slender to robust, brownish red or violet; flowers ± lax, over most of stem	Slender, purple; flowers ± lax, over the upper part of the stem	Slender to robust, purple or pinkish-brown; flowers ± dense, over the upper part of the stem	Slender, purplish to reddish; flowers lax, over most of stem
Floral bracts	10–12 mm, markedly shorter than the corolla	10–15 mm, slightly shorter than the corolla	13–16 mm, ± Equalling the corolla	14–17 mm, markedly exceeding the corolla	6–22 mm ± equalling to exceeding the corolla
Calyx	5–7 mm, segments fused, unequal (rarely entire)	10–15 mm, segments free	9–12 mm, segments fused, unequal	10–13 mm, segments free, unequal	6–14 mm, entire or segments fused, unequal to subequal
Corolla	10–15 mm, pale yellow with faint reddish veins	15–18 mm, cream with pink to violet veins	17–20 mm, cream with purple veins	16–25 mm, cream with purple veins	10–18(20) mm, cream with purple veins
Corolla dorsal line	Strongly cernuous to geniculate	Smoothly-curved	± erect, straight	± erect, straight	Smoothly-curved
Corolla indumentum	Sparsely glandular hairy throughout or basally glabrescent	Glandular hairy, basally glabrescent	Densely glandular hairy, basally glabrescent	Glandular hairy, basally glabrescent or glabrous	Glandular hairy, basally glabrescent
Filaments	Sparsely hairy below, glabrous above; inserted 5 mm above the corolla base	Hairy below, glabrescent above; inserted 2–4 mm above the corolla base	Hairy below, glabrescent above; inserted 2–3 mm above the corolla base	Hairy below, glabrous above; inserted 3–4 mm above the corolla base	Glabrescent or sparsely hairy below; inserted 2–3 mm above the corolla base
Stigma (typically)	Red-orange	Reddish-purple	Violet	Pink to violet	Pink to violet

The closest morphological match to the plants thus far identified as O.amethysteasubsp.castellana in the Canary Islands is in our view, *O.calendulae*: a rather confused taxon that tends to grow on coastal cliffs, primarily parasitizing *Calendulasuffruticosa* Vahl aggregate, and related species (and possibly other Asteraceae). It is generally described as having a curved, pale-yellow corolla (although see *taxonomic remarks*, below). Indeed the plant under consideration in the Canary Islands has also been identified as *O.calendulae* (Marrero et al. 1995), notwithstanding the atypical host (*Andryala*). However the plant in Lanzarote has a markedly cernuous (more rarely geniculate) corolla not seen in any other known species in the Subsection Minores, as well as distinct colouration and a high filament insertion. On account of this unusual combination of morphological features, and marked distribution and ecology, we examined material from the Canary Islands to resolve this long-standing taxonomic confusion.

## ﻿Materials and methods

### ﻿Plant material

Plants were observed in the field in Lanzarote between 2020 and 2023, and then cultivated in 2023–2024 to ensure morphological characteristics were stable (i.e. not a product of environment). Seeds were collected by the second author (MHG) from a population near Haría (29°08'07.4"N, 13°30'18.2"W) and sent to the first author (CJT) who cultivated the plant at the University of Oxford Botanic Garden on 10 potted specimens of *Andryalaperezii* grown under glass (ambient light, 15–20 °C). Seventeen spikes of *Orobancheandryalae* emerged in April, 4 months after planting, and each was measured and examined to inform the type description. Specimens were then compared with *O.minor* (growing spontaneously at the University of Oxford Botanic Garden) because this is the most widespread and common species in the Subsection Minores and co-occurs (at least on Lanzarote) with *O.andryalae*.

## ﻿Results

### ﻿ Orobanche andryalae

Taxon classificationPlantaeLamialesOrobanchaceae

C.J.Thorogood, M. Hernández González, Rumsey & Reyes-Bet. sp. nov.

9D4999B2-BB8A-5274-9E99-14F6898766F1

urn:lsid:ipni.org:names:77356942-1

#### Description.

Stems 6–16(25) cm, glandular-hairy, pale orange to light reddish-brown. Stem scarcely swollen below; subterranean bracts broadly triangular, yellow; those above (reduced leaves) rather sparse, brown, 8–15 mm. Flowers 5–15(20), arranged on the upper quarter or third of the stem, lax. Bracts 10–12 mm, rather shorter than the corolla, broadly triangular, brown, glandular-hairy. Calyx 5–7 mm with segments fused, strongly unequal (rarely entire), not exceeding the corolla tube. Corolla 10–15 mm, pale yellow with faint reddish veins and scattered glandular hairs, strongly cernuous when mature, remaining so in fruit, sometimes abruptly geniculate; upper lip bilobed; lower lip 3-lobed, the lateral lobes slightly exceeding the central; all lobes minutely-toothed. Filaments sparsely hairy below, glabrous above; inserted conspicuously (c.5 mm) above the corolla base; anthers ± glabrous. Stigma lobes touching, mid to dark red-orange.

#### Type.

Lanzarote, Canary Islands, Haría (29°08'07.4"N, 13°30'18.2"W); material grown from seed at the University of Oxford Botanic Garden, Oxford, United Kingdom; April 22, 2024. (holotype ORT 48576!), (isotype OXF! Barcode 00227715O).

#### Distribution, ecology and IUCN Red List status.

We examined *O.andryalae* in two locations on the island of Lanzarote, with five specimens at a population near Yé (29°11'46.1"N, 13°29'34.1"W), and about 40 in the other near Haría (29°08'07.4"N, 13°30'18.2"W) in 2020, 2021 and 2022. The plant was also observed in the Valle de Guerra and Teno regions of north Tenerife. Because *O.andryalae* is an annual, and based on observations of related species, we anticipate that numbers may fluctuate markedly from year to year (Rumsey and Thorogood 2023). *Orobanche* seed banks can, however, remain viable for decades (Rumsey and Thorogood 2023). Based on our current observations the species is likely to qualify for a threat status because of its restricted distribution, few locations and very low observed numbers and given that it co-occurs with its narrowly endemic host, *Andryalaperezii* on Lanzarote, and on *A.pinnatifida* on Tenerife (records on other endemic Asteraceae including *Asteriscusintermedius* (DC.) Pit. & Proust, *A.sericeus*, and *Crepiscanariensis* (Sch.Bip.) Babc. ex Jenkins require further investigation). *Andryalaperezii* – the predominant host, is locally common within its restricted range (Ferreira et al. 2014) and further parasite populations are to be expected on Lanzarote (the apparent stronghold for the parasite). *Andryalaperezii* also occurs on Fuerteventura, where we have observed *O.andryalae* (but here it was recorded on *Asteriscussericeus*; this too, requires further examination). *Andryalapinnatifida*, a recorded host in Tenerife, occurs across the western Canary Islands, again indicating *O.andryalae* may be under-recorded. We suggest that in the absence of long-term surveys, *O.andryalae* should, for now, be treated as DD (Data Deficient) (IUCN 2001).

#### Etymology.

*Orobancheandryalae* is named in accordance with its main host species, *Andryalaperezii*.

#### Taxonomic remarks.

**Figure 1.**
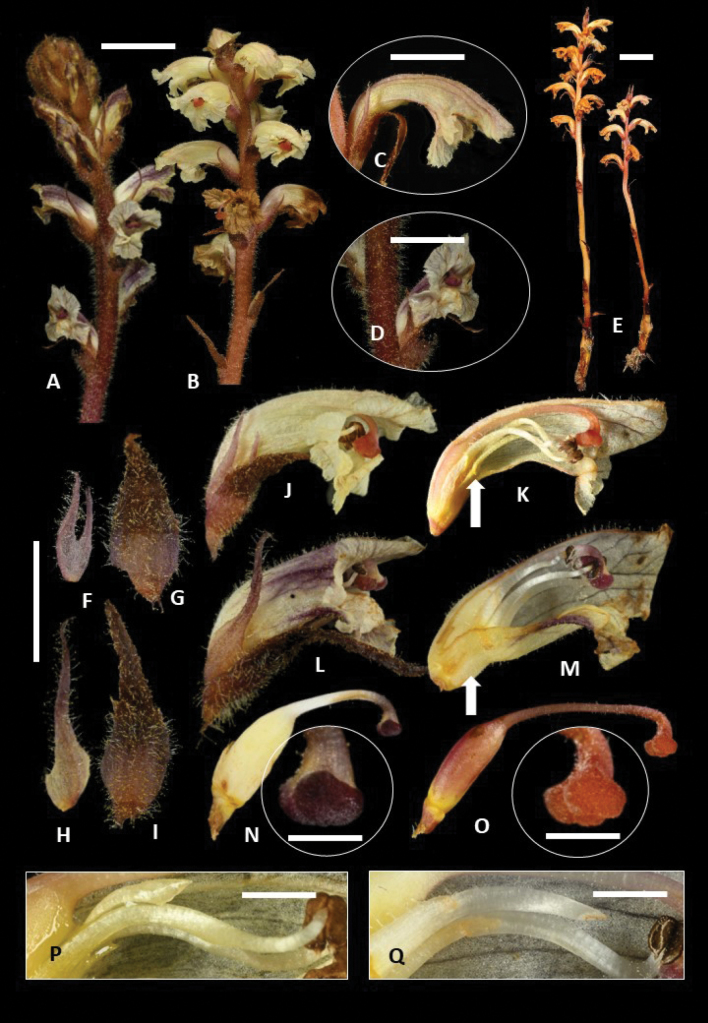
**A***Orobancheminor* habit **B***O.andryalae* habit **C***O.andryalae* corolla in profile **D***O.minor* corolla in profile **E***O.andryalae* whole plant **F, G** calyx and bract of *O.andryalae***H, I** calyx and bract of *O.minor***J, K***O.andryalae* corolla in profile with cross section; arrow indicates filament insertion point **L, M***O.minor* corolla in profile with cross section; arrow indicates filament insertion point **N** carpel and stigma of *O.minor***O** carpel and stigma of *O.andryalae***P** stamen of *O.andryalae***Q** Stamen of *O.minor*. Scale bars: 15 mm (**A, B**); 7.5 mm (**C, D**); 15 mm (**E**); 15 mm (**F, M**); 5 mm (**N–O**); 4 mm (**P, Q**).

**Figure 2.**
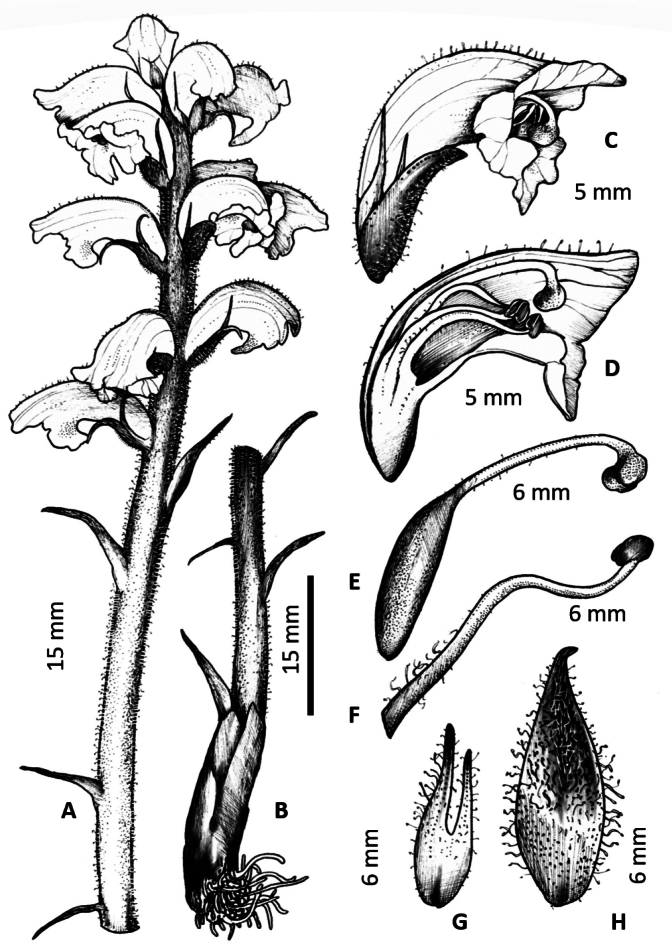
*Orobancheandryalae***A** habit **B** lower stem **C** corolla **D** corolla cross section **E** carpel **F** stamen **G** calyx lobe **H** bract.

**Figure 3.**
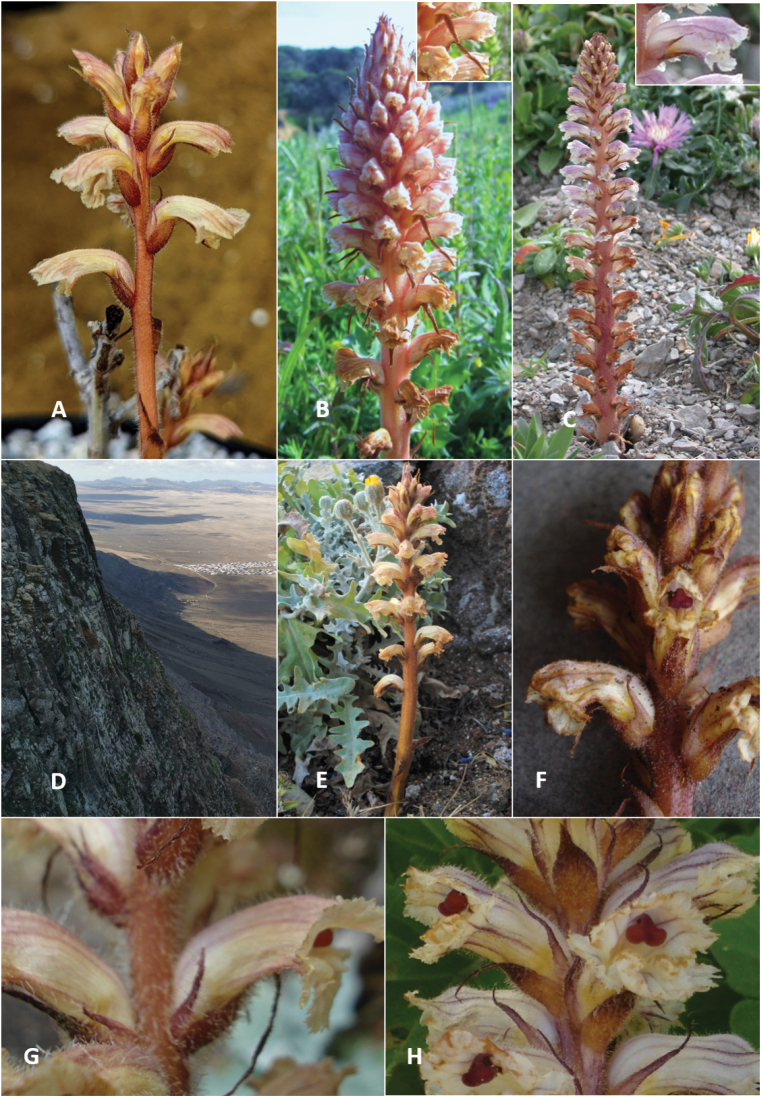
**A***Orobancheandryalae* in cultivation at the University of Oxford Botanic Garden **B***O.amethystea* in the Algarve, Portugal **C***O.calendulae* in the Algarve, Portugal **D** the habitat of *O.andryalae* in Lanzarote: volcanic cliffs (Famara area, northern Lanzarote) **E***O.andryalae* in Lanzarote, with its host plant *Andryalaperezii***F***O.andryalae* collected from Fuerteventura (putatively parasitising *Asteriscussericeus*) **G, H***O.andryalae* in northwest Tenerife growing on *Andryalapinnatifida* (the corolla dorsal line of the specimen in H is atypical in being less conspicuously cernuous).

**Figure 4.**
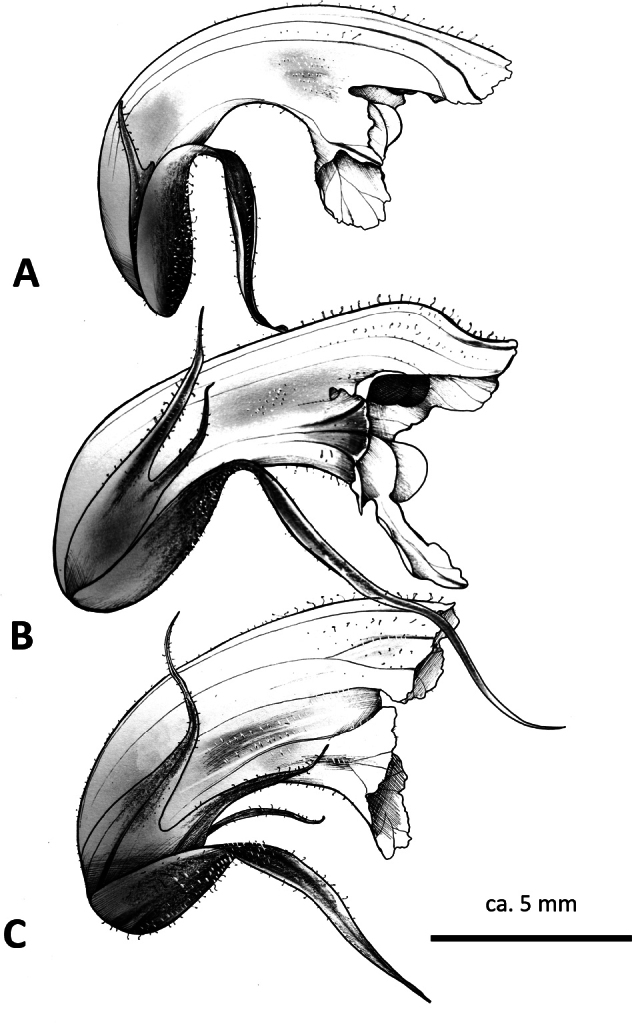
Corollas in profile **A***O.andryalae***B***O.amethystea***C***O.calendulae*. Note the scale is indicative only, as corolla length is variable.

*Orobancheandryalae* can readily distinguished by its strongly cernuous corolla (Figs 1C, 2C, 3A, 4A), coloration, filament insertion (Figs 1K, 2D), and distinct host and ecology. Importantly, these characteristics remain stable under cultivation (Fig. 3A). The plant is distinct from O.amethysteasubsp.castellana, which it is now clear does not occur in the Canary Islands, and we do not discuss further here. Rather, the closest taxon to *O.andryalae* appears to be *O.calendulae*, which is a somewhat confused taxon, originally documented from Algeria (Pomel 1874), and since recorded from Madeira, Morocco, Portugal and Spain (Chater and Webb 1972), and Tunisia (El Mokni et al. 2023). *Orobanchecalendulae* has marked host specificity and ecology, growing on relatives of the *Calendulasuffruticosa* aggregate on sea cliffs. Beck-Mannagetta (1930) also recognised a similar entity, *Orobanchemauretanica* Beck, mainly on the basis of distinct calyx characteristics: connate in *O.mauretanica* and entire, or bifid and free in *O.calendulae*. He described a variety of O.mauretanicathat he namedvar.calendulae, from the Algarve region of Portugal, which is parasitic on *Calendulasuffruticosa*. Greuter et al. (1989), however, synonymised *O.mauretanica* under *Orobanchecalendulae*, a decision that has been followed rather inconsistently; since that time, floras have also differed somewhat in their descriptions of *Orobanchecalendulae*. For example, both Flora Europaea (Chater and Webb 1972) and Flora Iberica (Foley 2001), describe equal calyx segments that are fused at the base; we note that the calyx segments in the holotype from Algeria are bifid but somewhat unequal; in the type description, they are reported to be entire or bifid (Pomel 1874).

The first author (CJT) has observed *Orobanche* populations on sea cliffs in the Algarve growing on *C.suffruticosa* (Fig. 3C, 4C) that may pertain to Beck’s O.mauretanicavar.calendulae (= *O.calendulae*). These plants possess pale orange stems, whitish corolla with violet pigmentation, reddish stigmas, apically filiform calyx lobes, and flowers arranged over most of the stem. These features appear to be consistent with the holotype of *O.calendulae* (Pomel 1874). But to complicate matters further, this plant (*O.calendulae* s.l.) co-occurs in the Algarve with *O.minor* (on various hosts), *O.litorea* Guss. (on *Plantago*), *O.balsensis* (J.A.Guim.) Carlón, M.Laínz, Moreno Mor. & Ó.Sánchez (on *Carlina*), and interestingly, *O.amethystea* (on *Eryngiumcampestre*) (Fig. 3B, 4B); Beck-Mannagetta also recognised a form of *O.mauritanica* on *Eryngiumcampestre* in the Algarve (‘forma dioristha’). Presumably this form in fact pertains to *O.amethystea*, but in the absence of material to examine, this remains a mystery. Again, this demonstrates the nontrivial historical confusion surrounding the taxa in this area, and the importance of examining a range of characters, as well as ecology and host identity when describing OrobancheSubsectionMinores.

Furthermore, we should note that populations of an unexamined entity in the Subsection Minores in Madeira also grow on *Andryala*, in this case *A.glandulosa* Lam. which is endemic to Madeira, Porto Santo and the Desertas. These plants have puberulent, yellowish stems, rather flat-backed corollas, long, apically filiform calyx lobes, and pinkish stigma lobes. Despite their Macaronesian distribution and host, they are clearly distinct from the Canary Island plant under consideration. They do, however, show a superficial similarity to *O.litorea*, and deserve further attention.

Notwithstanding the confusion surrounding the Subsection Minores, none of the taxonomic entities considered hitherto in this complex possess the stable combination of features we observe in *Orobancheandryalae* in the Canary Islands: a markedly cernuous corolla, high filament insertion and yellowish-orange colouration with a reddish stigma, and specificity for *Andryala* spp. on thermophilous volcanic substrates (Fig. 3D). In advance of a well-resolved phylogeny, it is important that taxonomic entities in the Subsection Minores are characterised morphologically and ecologically, to enable robust sampling and nomenclature.

### ﻿Key to the *Orobanche* species known to occur in the Canary Islands

Note that *O.amethystea* is not included as we believe all records of the plant in the Canary Islands pertain to *O.andryalae*.

**Table:** 

1	Corolla whitish, distally blue-purple	***Orobanchecernua***
–	Corolla whitish to cream with reddish or violet veins (not distally blue-purple)	**2**
2	Flowers fragrant, corolla whitish, tubular-campanulate, lobes with crenate margins	***Orobanchecrenata***
–	Flowers not fragrant, corolla cream to pale yellow, narrowly-tubular, lobes not crenate	**3**
3	Stigma typically yellow, parasitic mainly on ivy in woods	***Orobanchehederae***
–	Stigma pink or red-orange, not in woods	**4**
4	Corolla with an evenly-curved to almost straight dorsal line, filaments inserted 2–3 mm; various hosts, disturbed habitats	***Orobancheminor***
–	Corolla markedly cernuous (to geniculate), filaments inserted c. 5 mm; on *Andryala* spp. on volcanic substrates	***Orobancheandryalae***

## Supplementary Material

XML Treatment for Orobanche andryalae

## ﻿Additional information

### Author ORCIDs

Chris J. Thorogood https://orcid.org/0000-0002-2822-0182

Fred J. Rumsey https://orcid.org/0000-0003-4459-6769

Jorge Alfredo Reyes-Betancort https://orcid.org/0000-0003-0732-3219

### Data availability

All of the data that support the findings of this study are available in the main text.
